# CRISPR/Cas9 Epigenome Editing Potential for Rare Imprinting Diseases: A Review

**DOI:** 10.3390/cells9040993

**Published:** 2020-04-16

**Authors:** Linn Amanda Syding, Petr Nickl, Petr Kasparek, Radislav Sedlacek

**Affiliations:** 1 Laboratory of Transgenic Models of Diseases, Institute of Molecular Genetics of the CAS, v.v.i, 252 50 Vestec, Czech Republic; 2 Czech Centre for Phenogenomics, Institute of Molecular Genetics of the CAS, v.v.i, 252 50 Vestec, Czech Republic

**Keywords:** rare disease, CRISPR/Cas9, epigenome editing, transcriptome editing, genomic imprinting, Angelman syndrome, Prader-Willi syndrome, transient neonatal diabetes mellitus, Silver-Russell syndrome

## Abstract

Imprinting diseases (IDs) are rare congenital disorders caused by aberrant dosages of imprinted genes. Rare IDs are comprised by a group of several distinct disorders that share a great deal of homology in terms of genetic etiologies and symptoms. Disruption of genetic or epigenetic mechanisms can cause issues with regulating the expression of imprinted genes, thus leading to disease. Genetic mutations affect the imprinted genes, duplications, deletions, and uniparental disomy (UPD) are reoccurring phenomena causing imprinting diseases. Epigenetic alterations on methylation marks in imprinting control centers (ICRs) also alters the expression patterns and the majority of patients with rare IDs carries intact but either silenced or overexpressed imprinted genes. Canonical CRISPR/Cas9 editing relying on double-stranded DNA break repair has little to offer in terms of therapeutics for rare IDs. Instead CRISPR/Cas9 can be used in a more sophisticated way by targeting the epigenome. Catalytically dead Cas9 (dCas9) tethered with effector enzymes such as DNA de- and methyltransferases and histone code editors in addition to systems such as CRISPRa and CRISPRi have been shown to have high epigenome editing efficiency in eukaryotic cells. This new era of CRISPR epigenome editors could arguably be a game-changer for curing and treating rare IDs by refined activation and silencing of disturbed imprinted gene expression. This review describes major CRISPR-based epigenome editors and points out their potential use in research and therapy of rare imprinting diseases.

## 1. Introduction

### 1.1. CRISPR Epigenome Editors

The current course of genome engineering is set by tools derived from the bacterial immune system referred to as CRISPR/Cas9 (Clustered Regularly Interspaced Short Palindromic Repeats/CRISPR-associated protein 9). The system uses a ribonucleoprotein complex consisting of a short RNA molecule, a guide RNA (gRNA) and protein with nuclease activity Cas9 protein. The canonical CRISPR/Cas9 system acts as a site-specific nuclease, targeting a substrate DNA sequence, dictated by gRNA. Nuclease activity is executed by two Cas9 cleavage domains, RuvC (endonuclease domain termed after E.coli protein associated with DNA repair) and HNH (endonuclease domain termed after characteristic histidine and asparagine residues), which together mediate a double-strand break (DSB) [1,2]. This mechanism has been broadly used for generating disease or transgenic models [3,4,5]. However, further improvement of the canonical CRISPR/Cas9 system led to more accurate genetic manipulations, exceeding the ‘double-cut-based’ editing.

An example of a more specific editor is the Cas9 nickase, a modified Cas9 protein with an inactivation mutation in one of the DNA cleavage domains, which results in single-strand breaks (SSB). The use of two adjacent nickases instead of the canonical Cas9 protein has proven to be more precise, with lower off-target effects and increased probability of the cell shifting towards homology-directed repair [6,7]. Further mutations of RuvC and HNH domains gave rise to a catalytically inactive “dead” Cas9 (dCas9). Establishment of dCas9 has further extended the application of CRISPR technology to gene regulation editing and opened a new venue to the understanding of diseases etiologies linked to epigenetic dysregulations [8,9,10].

Epigenetics, literal translation “on top of the genetics”, involves mechanisms of gene regulation without changing the DNA sequence. The most critical processes participating in gene regulation are DNA methylation, histone modification, and chromatin remodeling [11]. Epigenetic machinery is controlled by groups of enzymes which are divided into three groups: writers, erasers, and readers.

DNA methylation is an epigenetic modification playing a crucial role in many regulatory processes. It is involved in regulation of transcriptional gene expression, genomic imprinting, X inactivation, silencing of mobile elements, and maintenance of genome integrity [12]. In the mammalian genome, DNA methylation or demethylation occurs at CpG sites, which are distributed throughout the genome, mainly in CpG-rich regions such as CpG islands. CpG islands can be part of promoters and distal regulatory elements where their methylation status contributes to gene transcriptional regulation. DNA methylation serves as a signal for the recruitment of epigenetic modifiers affecting the histone code or chromatin remodeling factors and often has a repressive effect [13,14].

The biochemistry of DNA methylation is based on the enzymatic addition of a methyl group to cytosine. This process is catalyzed by the “writer”—DNA methyltransferases (DNMTs). DNMTs are divided according to their function. Maintaining DNMT1 is responsible for methylation of hemimethylated DNA after replication. Methyltransferases DNMT3A, DNMT3B, and DNMT3L (co-factor DNA methyltransferase without enzymatic activity) are crucial for de novo DNA methylation, including the establishment of imprinting or gene silencing during embryonic development [15,16].

Removal of the methyl group from methylated cytosine (5mC) is either passive or active. The passive process of demethylation is replication-dependent and occurs throughout replication when a new DNA strand is not re-methylated yet. The active demethylation is a replication-independent process coupled with the conversion of methyl-cytosine to cytosine through oxidation or deamination [17]. Major ‘erasers’ or enzymes involved in the active demethylation are dioxygenases from the ten-eleven translocation family (TET1, TET2, and TET3) [18]. TET enzymes oxidize 5mC and produce 5-hydroxymethylcytosine (5hmC) intermediate, which is then oxidized to 5-formylcytosine (5fC) and 5-carbocylcytosine (5caC). These two intermediates are recognized and excised by thymine DNA glycosylase (TDG) [19]. The deamination pathway converts 5mC or 5hmC into 5-hydroxymethyluracil (5hmU) by the activation-induced deaminase (AID) and apolipoprotein B mRNA-editing enzyme complex (APOBEC), and 5hmU is then removed by uracil DNA glycosylase (UNG) [20,21].

Another regulatory process is histone modification, which involves chemical alternations on unstructured ends of histone proteins. Histone modification, essential for gene regulation, is conferred by methylation and acetylation on histones located in the vicinity of promoters or enhancers. The most studied histone modifications associated with gene activation are methylation of lysine 4 on histone 3 (H3K4) and lysine 36 (H3K36), or acetylation of lysine 9 (H3K9) and lysine 27 (H3K27) [22,23,24]. In contrast, modification, such as methylation of lysine 9 on histone 3 (H3K9), lysine 27 (H3K27) or lysine 20 (H3K20), are often linked to transcriptionally-repressed genes [22,25,26]. Enzymes responsible for histone modifications are designated as “writers” (adding methyl and acetyl residues), histone acetyltransferase (HAT) and histone methyltransferase (HMT), and “erasers” (removing the residues) histone demethylase (HDM) and histone deacetylase (HDAC) [27,28].

The epigenetic code is read by “readers”, molecules perceiving the information encoded in the epigenetic code and recruiting other factors influencing the epigenetic landscape. Readers dispose of specialized domains through which they recognize changes in chromatin marks and attract a particular group of proteins, activators or repressors, depending on the epigenetic context [28]. Examples of DNA methylation readers are proteins MeCP2, MBD1–6 or SETDB1/2. In the case of the histone code, for instance, methylation is read by HP1 or Polycomb proteins, and acetylation by CREBBP or EP300 proteins and many others. The readers potentiate gene activation or repression and are a crucial part of epigenetic machinery utilized by epi-editor systems [28,29].

The field of genome engineering has adopted writers, erasers and, indirectly, readers, and developed new epigenome editing tools, enabling modulation of gene expression without altering genetic information.

### 1.2. Genomic Imprinting, Rare Imprinting Diseases, and Epigenome Engineering

Genomic imprinting, a term describing the parent-of-origin expression of specific genes was first described in 1984 when it was shown that both the maternal and paternal genome were crucial for normal development of mouse embryos [30,31]. Since then, over 150 imprinted genes have been verified in the murine genome, and approximately half of them have been found in humans [32]. Most imprinted genes have important placental and developmental functions. Increasing evidence has shown that they also regulate metabolism, stem cell function, sleep patterns, and feeding. The imprinted genes tend to be organized in clusters and 13 clusters have been identified so far. The clusters vary significantly in size from only a few genes to several megabases containing both maternally- and paternally-expressed genes [33]. These gene clusters are modulated by imprinting control regions (ICRs) that regulate the expression of the imprinted genes in cis [32]. The ICRs are methylated either maternally or paternally in the gametes by a robust mechanism involving transcription [34]. A methylated ICR is inactive, whereas an unmethylated ICR is active and alterations of the methylation on the ICRs lead to disrupted expression levels of the genes under its control. ICRs on the maternally inherited chromosomes are mostly hypermethylated as a mode of imprinting, the methylation is conferred by DNMT3A and its co-factor DNMT3L during oogenesis. The ICRs from the maternally inherited copies generally encompasses the promoters of large untranslated antisense transcript, overlapping protein-coding genes, when transcribed [35]. In male germ cells, these ICRs are fully unmethylated, allowing for the long non-coding antisense transcripts to be expressed, thus silencing the protein-coding genes, of which they overlap. The exact mode of action by which it silences the gene is still under debate [36]. Although the majority of the DNA methylated ICRs are acquired in the female germline, there are paternally-methylated imprinted loci described. The ICRs regulate the H19/IGF2, Rasgrf1 and Dlk1/Gtl2 loci [37].

The IGF2/H19 ICR is present on chromosome 11 and regulates fetal growth. Aberrations on this loci such as paternal hypomethylation of the ICR is present in the majority of patients with the imprinting disease Silver–Russell syndrome [38].

Disturbed expression of imprinted genes can occur either through genetic or epigenetic mechanisms. Genetic perturbation of imprinted genes can occur through mutations in the gene, duplications and deletions of larger segments. Uniparental disomy, a phenomenon where the chromosome is inherited from one parent-of-origin is also commonly occurring in diseases caused by imprinting defects. Epigenetic mutations cause aberrant expression via alteration in DNA methylation marks of ICRs in the imprinting clusters [39].

Imprinting diseases (IDs) are a group of rare congenital diseases that are caused by the aforementioned perturbations in imprinted genes. There are eight separate diseases known to us, but there are most likely more to be discovered and described [40]. Patients with rare IDs commonly carry intact genes but that are silenced through epigenetic mechanisms or by overexpression of imprinted genes. This taken together makes it evident that rare IDs have little benefit of first-generation CRISPR genome editing, relying on dsDNA breaks subsequently repaired through the error-prone pathway NHEJ or the low-frequency HDR pathway. Rather, they are in dire need of sophisticated editing of the epigenome to activate existing but silenced genes. In the following sections, we will describe the molecular genetics of four known rare IDs and the possible applications new generation CRISPR epigenome editors could offer.

## 2. CRISPR-Based Epigenome Editors (CRISPR Epi-Editors)

The epigenetic landscape can be manipulated by small molecules referred to as epigenetic drugs. These drugs target writers and erasers and alter or inhibit their function, causing changes in the epigenetic state [41]. Although epigenetic drugs represent promising treatment for cancer, cardiovascular, neurological diseases, and metabolic disorders, their effect is broad, and they lack locus specificity [42,43].

A more specific alternative to epigenetic drugs might be epigenome editing by CRISPR/dCas9, which enables locus-specific epigenome alternations [44]. CRISPR-based epigenome editors (CRISPR epi-editors) consist of dCas9 and epigenetic effector, fused or non-covalently bound to dCas9 [3,44,45,46,47]. This complex is navigated by gRNA to a target sequence in which vicinity the epigenetic landscape is edited by the effector. The effector is either activator or repressor of gene transcription depending on the origin of the effector. The effectors are derived from epigenetic writers and erasers, such as DNMTs, HATs, HMTs and TETs, HDM, and HDAC, respectively [10,48,49]. Therefore, compared to the small molecules, that can relatively easy penetrate tissues of interest, CRISPR epi-editors might have a problem to be efficiently delivered in vivo. The most promising delivery system for CRISPR epi-editors with prolonged expression is AAV (adeno-associated virus) vectors with a packaging capacity of ~5 kb [50]. In spite the size of the SpCas9-gRNA-effector (*Streptococcus pyogenes* Cas9) complex exceeds an average packaging limit, the effective in vivo delivery is achievable with smaller dCas9 variants, or a different, less immunogenic delivery systems, such as EVs (extracellular vesicles), carrying CRISPR epi-editor plasmids or viral vectors [50,51,52,53,54]. Achieving the efficient delivery, high specificity, and non-immunogenicity represent the most crucial challenges standing before epigenome editing [55].

CRISPR epi-editors may be divided into four groups by their mode of action: chromatin reorganization, expression regulation, covalent histone and DNA modification [3,10,49,56]. Current research employs mainly the last three groups. Expression regulators, referred to as CRISPR activation (CRISPRa) and CRISPR interference (CRISPRi), use domains of transcriptional activators or repressors which mediate recruitment or blockage of transcription factors affecting transcriptional machinery [10,45,46,57]. In contrast, epi-editors with catalytic domains responsible for covalent histone modifications or DNA methylation are actors with own enzymatic activity [58,59,60,61].

The following sections provide an overview of the most relevant CRISPR epi-editors and their prospects in research or treatment of mentioned IDs.

### 2.1. DNA De/Methylation Mediated by CRISPR Epigenome Editors

Knowledge of the molecular mechanisms linked to methylation and demethylation contributed to the development of epigenome editors. Catalytic domains of enzymes responsible for DNA methylation have been adopted by CRISPR technology and given rise to programmable epi-editors capable of editing DNA methylation.

The first programmable DNA methylation editors were based on a fusion of the catalytic residues of programmable DNA binding molecules, such as ZFN or TALEN [62,63,64,65]. CRISPR epi-editors are designed by similar principles, through fusion or non-covalent attachment of active domains to DNA binding molecules; in this case, dCas9 [60,66,67,68]. However, CRISPR epi-editors, in contrast to ZFN and TALEN based epi-editors allow inexpensive and easily programmable epigenome engineering with a possibility of large-scale throughput analysis [69].

The current research focused on epigenome editing through DNA methylation mainly takes advantage of DNMTs or TETs. As mentioned above, DNMTs enzymes add the methyl group to cytosine, which has a silencing effect [15,16]. Therefore, the DNMTs catalytic domains have been attached to dCas9 protein and produced a programmable silencing complex. In contrast, TETs, in combination with dCas9, have been used for demethylation leading to decondensation of chromatin and subsequent binding of transcription factors [16,60,67,70].

**Figure 1:**
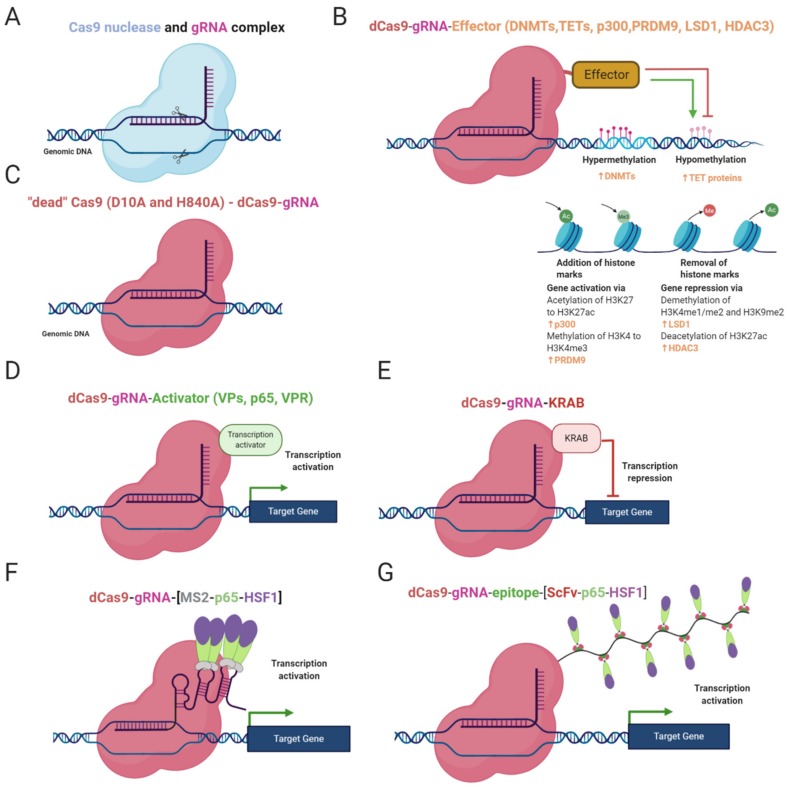
Epi-editor systems and their constitution. (**A**) Cas9 nuclease executing site-specific DSB; (**B**) dCas9 protein with effector domain of DNMTs or TETs or p300 or PRDM9 or LSD1 or HDAC3. DNMTs repress gene regulation through DNA methylation, TETs mediate demethylation of DNA and activate gene expression. p300 acetylates H3K27 and PRDM9 adds a third methyl residue on H3K4, with both effectors promoting gene expression. LSD1 removes methyl groups from H3K4me1/2 and H3K9me2, and HDAC3 deacetylates H3K27ac, with both modifications leading to repression of gene expression; (**C**) dCas9 protein with inactivation mutations, D10A and H84A in domain RvuC and HNH, respectively (**D**); CRISPR activator, dCas9 fused to distinct trans-activation proteins, such as VP64, p65, Rta; (**E**) CRISPR interference complex, dCas9 with KRAB repressing gene expression (**F**) CRISPRa, synergistic activation modulator (SAM) tethering trans-activating molecules (p65 and HSF1) on RNA scaffold through MS2 proteins. (**G**) CRISPRa, gene activating SunTaq system, consisting of dCas9 with repetitive peptide epitopes bound by single-chain variable fragment antibodies (ScFv) fused to trans-activation proteins (p65 and HSF1).

DNA methylation status can be edited by gRNA/dCas9-effector complex where the effectors are often DNA methyltransferases, mostly DNMT3A and DNMT3L (Figure 1B). DNMT3L lacks a catalytic domain mediating DNA methylation but enhances methylation by DNMT3A [16,60]. The effector can be either fused to the dCas9 protein through a linker or attached to RNA aptamers (e.g., MS2, com, PP7) or repetitive peptide epitopes via binding proteins (RNA aptamer binding proteins, e.g., MCP, COM, PCP; repetitive peptide epitopes binding proteins, e.g., single-chain variable fragment (ScFv) antibody). The advantage of the attached effector system is the potential recruitment of multiple copies of the effector, leading to a more robust change in methylation status (Figure 1F,G) [60,66,67,68]. Epi-editors with DNMT catalytic domains modify CpG-rich loci in the manner described above, leading to silencing of gene expression and chromatin rearrangements [15,16]. Locus-specific DNA methylation is enhanced while combinations of epi-editors are used, for instance, triple recruitment of DNMT3A, DNMT3L, and KRAB domains [66,71].

It has been reported that site-specific DNA methylation is achievable with the dCas9-DNMT complex. However, multiple studies have pointed out a higher efficiency of site-specific DNA methylation when DNMTs are multimerized. For example, Huang et al. (2017) showed that tethering of DNMT3A domains via SunTag system can lead to more robust and precise locus-specific DNA methylation due to a multimerization of DNMTs [66]. A similar synergistic effect has been shown by Amabile et al. (2016) after combinatorial use of DNMT3A, DNMT3L, and KRAB, fused to ZFP, followed by Stepper at al. (2016) with DNMT3A and DNMT3L fused to dCas9 [60,71].

In the past, DNA demethylation epi-editors were TALEN- or ZFP-based and fused with TET1 catalytic domain. Afterwards, CRISPR technology came and took over the locus-specific DNA demethylation [47,64,65,67,70]. CRISPR demethylation editors are structured in a similar way as DNA methylation systems but employ catalytic domains (CD) of functionally antagonistic enzymes, such as TET1 or TET3 (Figure 1B) [67,71]. Catalytic residues can be fused to dCas9 or attached to the gRNA/dCas9 complex via RNA aptamers or peptide repeats as described above [47,67,71].

TET catalytic domains have been proven effective in regards of site-specific DNA demethylation. Xu et al. (2016) have demonstrated demethylation of genes with CpG-rich promoters mediated by TET1-CD tethered via gRNA scaffold to dCas9 in vitro [67]. Furthermore, the potential of dCas-TET constructs was also proven in vivo by Xu et al. (2018). The Xu group targeted dCas9-TET3CD complex to genes essential for suppression of renal fibrosis. After treatment of affected mice, researchers observed elevated expression of targeted genes, together with decreased promoter methylation and reduced fibrogenesis in kidneys [70]. These studies imply that CRISPR epi-editor systems with TET catalytic domains are capable of effective demethylation in a locus-specific manner.

### 2.2. Histone Modifications by CRISPR Epi-Editors

Targeting the histone code represents another approach of intentional manipulation of the epigenetic landscape. Thus, catalytic domains of histone code maintaining enzymes have been adopted for epigenome editing. For instance, catalytic domains of PRDM9 (HMT), LSD1 (HDM), p300 (HAT), or HDAC3 (HDAC) have been fused to ZF, TALE, or dCas9 proteins.

Each of the listed domains is involved in the modification of lysine residues on histone 3 as a group of the crucial marks affecting gene regulation. Dead Cas9 fused to the SET domain of human PRDM9 mediates the addition of methyl groups to H3K4 and attaches the gene expression inducing mark, H3K4me3 (Figure 1B) [61]. The dCas9-LSD1 complex has an antagonistic effect and catalyzes the removal of the methyl group from H3K4me1/2 and H3K9me2, which has a repressive effect (Figure 1B). dCas9-LSD1 generally downregulates expression of the target gene through histone modification in a site of the cis-regulatory element or promoter [72,73]. Gene regulation can also be regulated through histone acetylation. The fusion of dCas9 and p300 catalytic domain enables the locus-specific addition of acetyl groups on H3K27, resulting in transcriptional activation (Figure 1B) [58]. Partially in contrast to the dCas9-p300 complex, dCas9 fused to HDAC3 can either repress or promote transcription by removing the acetyl group on H327ac depending on the chromatin context (Figure 1B) [74]. The precise application of histone code targeting epi-editors has the potential to be used for deciphering the basis of rare diseases caused by dysregulation of histone modifications.

### 2.3. Gene Regulation by CRISPRa and CRISPRi Systems

CRISPR activation and CRISPR interference systems are CRISPR-based epi-editors consisting of dCas9 and an effector domain. Unlike the previous epi-editors, the effector domains of CRISPRa and CRISPRi are not catalytically active [9,48]. The mode of action is the recruitment of transcription promoting or repressing molecules, depending on features of the effector. CRISPRa system employs several activation factors, for instance, the transactivation domain of NF-κB p65 subunit (p65AD), herpes simplex viral protein 16 (VP16), four tandem copies of VP16 (VP64), or ten tandem copies of VP16 (VP160), (Figure 1D,F,G) [29,48,75,76].

The pursuit for more significant activation led to the development of tripartite target gene activator dCas9-VPR, consisting of dCas9 and tethered V64, p65AD, and Rta (Epstein–Barr virus trans-activator) proteins (Figure 1D). The system was proven to be a potent transcriptional activator, especially when multiplexing gRNAs along the promoter of interest [46].

Another approach for increasing the efficiency of activation is the use of synergistic activation mediator (SAM), using modified gRNA as a scaffold. As noted earlier, the scaffold is a tandem of aptamers (MS2, com, PP7) which are bound by RNA-binding proteins fused to a functional protein. In this case, functional proteins are p65AD, heat shock factor 1 (HSF1) with MCP proteins (Figure 1F). This scaffold can associate with dCas-V64 fusion protein and give rise to more efficient transcription activator [68,77].

An alternative to SAM is the SunTag system, tethering multiple V64 molecules through ScFv antibodies (Figure 1G) [78]. Although VPR, SAM, and SunTag systems are considered the most potent, they still cannot achieve maximal activation. Their high rate of performance is dependent on multiplexing gRNAs along the promoter, and their efficiency differs depending on the cell line, selected gRNAs, locus and chromatin environment [3,78]. An elegant solution for multiple gene regulation has been shown by Campa et al. [79]. The approach stands on expression of a relatively compact cassette consisting of ddCas12a (DNase dead Cas12a from *Acidaminococcus* sp.) fused to an effector and array of gRNAs, where each gRNA can affect different gene. Although the size of the cassette is reduced compared to other broadly used epigenome editing systems, it exceeds packaging capacity of AAV vectors. Therefore, use of more than one vector is still necessary, but this system represents a first step towards a robust, large-scale epigenome editing in vivo [79].

In the case of target gene repression, it can be achieved by using CRISPR interference tools which are based on dCas9 protein fused to a repressor domain, silencing the target gene. The first generation CRISPRi consisted of a gRNA/dCas9 complex (Figure 1C). The complex was targeted to the promoter sequence and sterically blocked gene expression. Even though this simple system could decrease the expression of target genes by 99% in prokaryotes, its performance in eukaryotic cells has not been so robust, 60–80% [80]. To produce more efficient programmable gene repressor, domains of mammalian transcriptional repressor were adopted and fused to dCas9 protein. CRISPRi system-mediated gene repression has been attempted with various dCas9 fusion proteins. For instance, Gilbert et al. have tested three different repressive domains: KRAB (Krüppel associated box) derived from transcription repressor Kox1 protein (ZNF10) (Figure 1E), CS domain (Chromo Shadow) of heterochromatin protein 1 (HP1), and the WRPW motif of transcription factor Hairy and enhancer of split-1 (HES1). The results showed that KRAB fusion is the most effective in target gene repression [48]. Therefore, the combination of KRAB repressor and dCas9 has been extensively used in other studies until the present day [57,81].

Activator and repressor systems can be combined with histone code epi-editors resulting in an extension of their ability to activate or repress target genes not only through recruitment of transcription machinery on the promoter but also affecting cis-regulatory elements [82].

### 2.4. Delivery

Therapeutic applications of epigenome editing require safe, efficient, tissue-specific delivery and sustained inducible expression, all of which can be partially achieved with viral vectors. Although lentiviral or retroviral delivery systems are capable of carrying large transgenic cassettes, their immunogenicity, the potential to competent other viruses, and tendency to randomly integrate into the host genome make these systems less convenient for these types of therapeutic applications, even though attempts to reduce their integrative potential have been made [83,84]. In contrast, adenoviral vectors are non-integrative with transient expression and excellent transduction efficiency but with a risk of severe antigenicity [85]. According to many studies, potentially the least harmful vectors are derived from adeno-associated virus (AAV). Despite their low packaging capacity and transduction efficiency, these vectors are capable of delivering transgenes in a tissue-specific manner, with relatively long-term transient expression without significant pathogenicity effects [45,86,87].

Nonetheless, recombinant AAV serotypes with increased packaging capacity and the ability to cross the blood-brain barrier have been developed. These improvements make AAVs a serious candidate for the primary delivery system of epi-editors [88,89]. However, the capacity expansion is not sufficient enough for AAV particle to carry a whole epi-editor system. Therefore, multi-component, trans-splicing systems, or more compact orthologues of SpCas9 (1 366 aa) has been employed, e.g., dCas9 from *Neisseria meningitides* (NmCas9,1 082 aa) or *Staphylococcus aureus* (SaCas9,1 053 aa) [90,91].

The multi-component system has been used by Liao et al. where dCas9 and gRNA with the SAM complex were expressed separately from two and/or more vectors [45]. Furthermore, trans-splicing allowed delivery of sizable Cas9 protein which is divided into two vectors, and the RNA is put together and forms one functional Cas9 coding mRNA via RNA trans-splicing machinery [92]. Cas9 orthologues represent smaller versions of SpCas9 derived from different bacteria species. They are capable of reducing the size of the carried gene and open a possibility to be efficiently packaged into AAV particles [90,91].

An alternative to viral vectors are extracellular vesicles (EVs) which are lipid-bilayer particles naturally released from cells. EVs can be derived from autologous cells. Therefore, they have low immunogenicity and also the ability to target tissue or cell population of interest. Their affinity to specific tissues may be controlled through modification or removal of surface receptors [93]. A possible disadvantage of EVs is a short lifetime in vivo (up to 6 h) compared to AAV-mediated delivery (up to 2 days) [94,95]. It has been shown that cell-derived EVs enable efficient drug or gene delivery with minimal immune response. In addition, it has been successfully achieved to supply the tissue of interest with an AAV transgene cassette without an antigenic effect with so-called vexosomes, a combination of EVs and viral particles [54,96].

### 2.5. Inducible Systems

**Figure 2:**
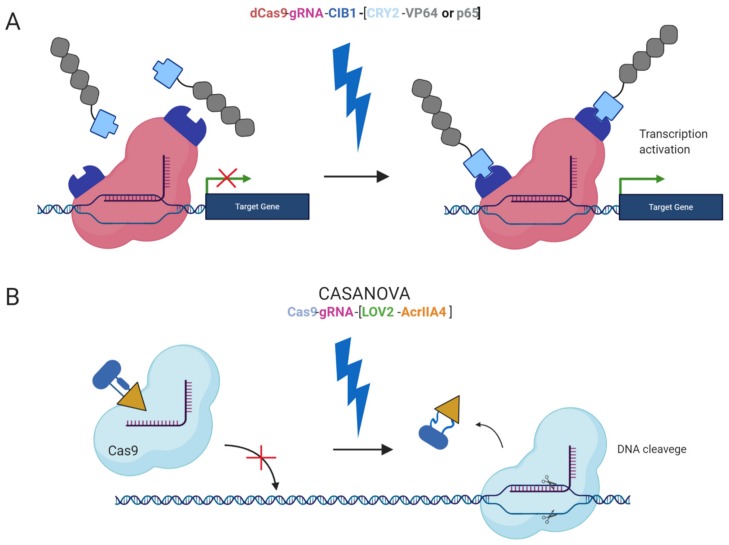
Light inducible CRISPR/Cas9 systems. (**A**) An inducible system based on blue light-dependent interaction between cytochrome 2 and CIB1 protein fused to CRISPRa components dCas9 and the effector (VP64 or p65). During exposure to the blue light two components are bound together and fully functional, upon removal of the blue light the complex is decomposed; (**B**) CASANOVA system, the blue light-inducible system controlling Cas9 nuclease activity via inhibitor LOV2-AcrIIA4. In the absence of the blue light, the inhibitor blocks Cas9 and prevents it from the binding the target sequence. In the presence of the blue light, the inhibitor is destabilized and released from Cas9 protein. Subsequently, Cas9 is active and executes DSB in the target locus when the blue light is removed the inhibitor binds back to Cas9.The chemically inducible systems are ligand-dependent. The interaction between the effector domain and dCas9 is conditioned by the presence of a ligand and two ligand-binding domains, linking two epi-editor components. Again, one of the binding domains fuses with the effector and the other with dCas9. In the presence of a ligand, both binding domains interact with the ligand and form a stable heterodimer resulting in the formation of an active epi-editor complex. Examples of ligand-binding domains are FK506 binding protein 12 (FKBP), and FKBP rapamycin binding protein (FRB) interacting together via rapamycin molecule (Figure 3A) [100], abscisic acid-induced dimerization of ABI and PYL1 domains (Figure 3B) [101], or gibberellin-induced dimerization of GID1 and GAI (Figure 3B) [101].

Furthermore, safer applications of the epi-editors have been examined by the utilization of inducible systems preventing the uncontrolled activity of the epi-editor cassette. Such an example is the optogenetic system, using two photoactivated binding proteins, cytochrome 2 (CRY2) and its interacting partner CIB1, to turn on epi-editor activity. In this case, the CRY2 domain is fused to the effector and CIB1 to dCas9 protein. The light-sensitive proteins, CRY2 and CIB1, interact when exposed to blue light. They activate the epi-editor by bringing together the effector and dCas9. The system is reversible, and after the removal of the blue light, the complex is destabilized and inactivated (Figure 2A) [97,98]. Another optogenetic system is CASANOVA (CRISPR–Cas9 activity switching via a novel optogenetic variant of AcrIIA4). Even though, this system has been used for gene editing it offers a new approach which can be adopted by epigenome editing system in the future. The CASANOVA system activates Cas9/gRNA complex upon blue light exposure. In the inactive state, the Cas9 complex is inhibited by a composite inhibitor AcrIIA4-LOV2, in which AcrIIA4 is an actuator derived from *Listeria monocytogenes* prophage fused to photosensor-LOV2 domain from A. sativa phototropin-1. When the inhibitor is exposed to blue light, it changes its conformation and allows the Cas9/gRNA complex to function. Upon removal of blue light, the inhibitor acquires its repressive conformation and binds back the Cas9/gRNA complex (Figure 2B) [99].

**Figure 3:**
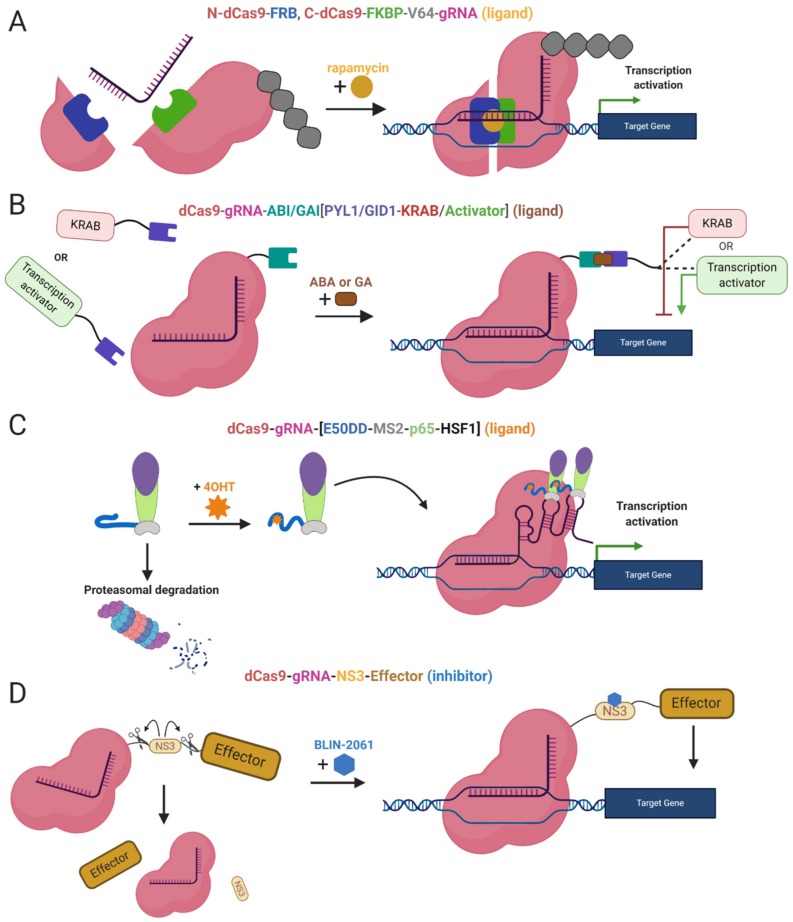
Chemically Inducible Epi-editors Systems. (**A**) Split dCas9-VP64 complex with one ligand-binding domain (LBD) at N-terminus of dCas9 and second LBD at C-terminus. Both LBDs bind a ligand (rapamycin, yellow) and bring together both halves of dCas9, resulting in the formation of functional gene activation complex; (**B**) Inducible system using phytohormones and phytohormone binding domains ABI or GAI fused to dCas9, and PYL1/GID1 fused to the effector-activator (VPR) or KRAB. The interaction via a ligand (abscisic acid or gibberellin) activates the epi-editor complex; (**C**) Inducible SAM system with a destabilized domain of estrogen receptor 50 (ER50DD). In the absence of a ligand-4OHT (4-hydroxytamoxifen), ER50DD protein is destabilized and leads the whole ER50DD-MS2-p65-HSF1 to proteasomal degradation, once the ligand is present it binds ER50DD and stabilizes it. Then complex is not degraded and therefore capable of interacting with RNA aptamers a form functional activation complex. (**D**) Inducible system with proteolytic cleavage. A part of the linker, between dCas9 and effector domain, is NS3 protein, protease from hepatitis C virus that cleaves peptide bonds in its vicinity. When NS3 is active, it cleaves the linked and abrogates the function of the epi-editor complex. The protease can be blocked by inhibitorBLIN-2061, leading to restoration of the epi-editor and its activity. The effect of ligands or inhibitors in the system mentioned above is reversible. After the inhibitor/ligand is diluted or metabolized, the chemical epi-editor systems are inactivated.

Moreover, other systems use ligands as inhibitors of proteolytic cleavage of a linker between dCas9 and the effector domain. The induction stands on the protease domain derived from the hepatitis C virus, ligand-inhibited NS3 protease. The protease domain connects dCas9 and the effector, the linker is cleaved unless the NS3 inhibitor is present. The inhibitor binds the NS3 domain and prevents separation of dCas9 from the effector (Figure 3D) [102]. A similar system takes advantage of proteasomal degradation, ligand-(Z)-4-hydroxytamoxifen (4OHT) binds a destabilized domain of the estrogen receptor fused to the effector domain and stabilizes it. In the absence of 4OHT, the whole effector complex is degraded in the proteasome. Thus, stabilization prevents degradation and the dCas9-effector complex is assembled and activated (Figure 3C) [103].

In conclusion, epi-editors represent a potent tool for gene regulation. Their combinatorial use provides a possibility of activation and repression of target genes at the same time without altering DNA or RNA sequence. They can compete with commonly used approaches such as RNAi or cDNA rescue with an advantage of higher, specificity, repression of all transcription variants or elevating the level of protein in isoform independent manner [104,105]. However, to make epi-editor systems safe for therapeutic purposes, many challenges must still be overcome—for instance, effective and non-immunogenic delivery with subsequent sustainable and tunable activity. Although off-target acting of epi-editor might seem less harmful compared to Cas9 gene editing, it is essential to make the epigenome editing as precise as possible due to the lack of complete knowledge of epigenome regulation. Therefore, the current research of epigenome editors aims for overcoming these challenges and possibly opens new avenues for treatment of diseases caused by epigenetic dysregulation.

## 3. Rare Imprinting Diseases and Therapy

Rare ID are in dire need of sophisticated editing of the epigenome to activate silenced genes. In the following sections, we review the molecular genetics of four rare IDs and how new-generation CRISPR epigenome editors could offer in terms of editing (Table 1).

### 3.1. Angelman Syndrome

Angelman Syndrome (AS) is a rare neurodegenerative disease linked to the imprinted chromosomal region 15q11.2–13q [106]. Main features of AS include severe mental retardation, epileptic seizures, gait ataxia, sleep disturbances, and a fascination for water [107]. The disease genetics are comprised by mainly four genetic etiologies (i) de novo interstitial deletion of 15q11.2–13q on the maternal chromosome, spanning approximately 4 Mb in total [108]; (ii) paternal uniparental disomy, both regions are paternally inherited thus exhibiting paternal expression only [109]; (iii) imprinting defects due to incomplete or faulty epigenetic modification of the ICRs necessary for correct regulation [110]; and (iv) point mutations on the brain-specific paternally imprinted *Ube3a* gene, encoding a ubitiquin E3 ligase targeting substrate proteins for proteasomal decay. This gene has been proven to be solely capable of causing AS, labelling it as the AS gene [111,112].

**Figure 4:**
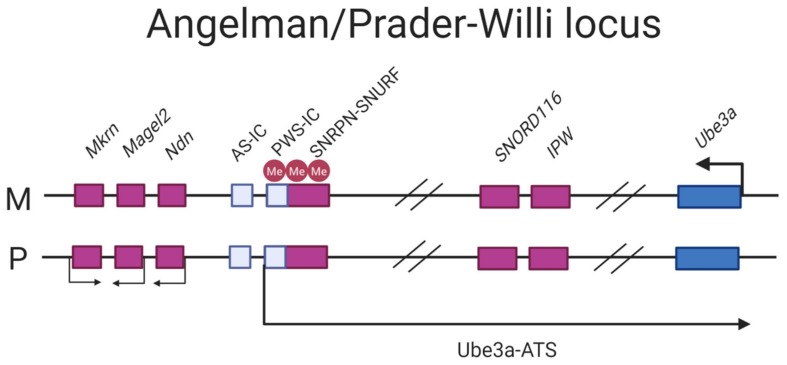
Schematic of the AS/PWS locus. The pink filled boxes: paternally expressed genes, blue filled boxes: maternally-expressed genes. The PWS/IC located in the promoter/exon 1 region of *SNRPN/SNURF* is hypermethylated on the maternally inherited chromosome thus silencing transcription of the Ube3a-ATS, allowing Ube3a to be expressed. The paternally-inherited PWS-IC is hypomethylated thus expressing the transcript, silencing *Ube3a*.

The mode of imprinting differs maternally and paternally in the locus. The maternally-inherited locus is associated with hypermethylation, with the imprint established in the gametes [113,114]. The paternal mode of imprinting is associated with hypomethylation of the region where silencing of the *Ube3a* gene is mediated by an antisense transcript (further referred to as Ube3a-ATS) that blocks the expression of the paternal copy in cis [115]. The transcription start site (TSS) of Ube3a-ATS initiates at the promoter/exon 1 region of the maternally imprinted gene *SNRPN* which is fully CpG methylated in the maternal copy and completely lacks methylation on the paternal one, rendering it transcriptionally active (Figure 4) [36].

Ameliorating phenotypes associated with AS can be achieved by solely reinstating the expression of *Ube3a*. As recently demonstrated, pharmacological reinstatement of the paternal copy of *Ube3a* rescued cognitive defects in a murine mouse models and has proven to fully restore hippocampal synaptic plasticity [116,117,118]. The topoisomerase I inhibitor topotecan have significantly increased the paternal *Ube3a* expression, but its lack of specificity together with its toxicity are limitations that do not allow its efficient use in the disease treatment [117]. Anti-Ube3a-ATS oligonucleotides, administered via intracerebroventricular injections were tolerated as well as it provided the specificity that topotecan lacks but is limited by its transient nature, only allowing for Ube3a-ATS silencing up to four months [118]. Viral vectors carrying mouse *Ube3a* has been attempted to rescue AS by delivery into the hippocampus of an AS mouse. This study demonstrated rescue in hippocampus-dependent learning and memory but no alteration in the phenotypes, including movement deficits [119]. Further attempts to activate the silenced copy were conducted by providing AS patients with pro-methylation dietary supplements during one year, with the rationale that an increase of global DNA methylation should allow for Ube3a-ATS silencing; however, this study was not successful [120]. As discussed by Bi et al. (2016), for un-silencing of the paternal *Ube3a* to be rendered as a successful therapeutic option the effect should be long-lasting, non-toxic and specific, which has not yet been achieved by the efforts mentioned above [121].

The novel CRISPR epigenome editors could putatively provide a solution to previous limitations in regards to specificity, longevity and toxicity. For instance, programming gRNA to guide the catalytically inactive dCas9 tethered with DNMT3A for the CpG islands at the TSS for Ube3a-ATS should mimic the maternal inactive methylation pattern, associated with increased *Ube3a* expression. As discussed in by Silva-Santos et al. (2015) there is a window for improving motor-function deficits that does not extend beyond the postnatal stage in development [116]. For ameliorating cognitive deficits, it seems to be a window closing much earlier. However, at a cellular level the plasticity of the hippocampal neurons can be rescued later on as no critical window seems to exist [116]. How to overcome the limitations caused by critical time-windows and which the best mode of delivery of treatments to patients would be, are two open questions for CRISPR based AS therapy. However, the CRISPR technology is continuously surmounting pitfalls such as off-target effects and delivery methods are being refined, preventing thus unspecific methylation and toxic reactions in the host. Furthermore, DNA methylation by dCas9-DNMT3A in cell lines has been proven to be stable and persisted through mitotic divisions [122]. Altogether, this points towards that CRISPR epigenome editing should be considered in the treatment of AS.

### 3.2. Prader–Willi Syndrome

Prader–Willi Syndrome (PWS) is linked to the locus 15q11–13q (Figure 4), as described for AS, distinguishing itself from AS by its clinical manifestation and parent-of-origin aberrancy. The majority of PWS patients harbor a 6 Mb or 5.4 Mb deletion, referred to as a type I and type II deletion on the paternal chromosome, respectively. Approximately 20–30% have maternal UPD; additional 1–3% have imprinting disorders leading to silencing of paternally expressed genes [123]. In contrast to AS where one main disease-causing gene has been confirmed, there are 15 genes in the PWS critical region [111,124]. However, evidence from collective efforts in deciphering the molecular genetics of PWS is suggesting that the C/D box snoRNA cluster SNORD116, expressed from its host transcript 116 HG, might be the key player in PWS [125]. C/D box snoRNAs are small nuclear RNAs that methylates ribosomal RNAs. Nevertheless, the SNORD116 cluster is considered non-canonical as it has no ribosomal RNA target and the function is largely unknown [126].

Additionally, sequencing of five patients with microdeletions has further narrowed the PWS critical region to 91 kb encompassing three non-coding genes; SNORD115, SNORD116, and IPW [127,128,129,130,131]. Where the loss of paternal inheritance leads to severe neuroendocrine and physical dysfunctions [132]. The full PWS phenotype is characterized by severe hypotonia with feeding difficulties in the first years of life that later progresses to hyperphagia, often leading to morbid obesity [133]. Moreover, PWS patients also exhibit hypogonadism, short stature, mild mental retardation, and psychotic behavior in adult life [134]. As the endocrine system is arguably the most affected system in PWS patients, growth hormone therapy ameliorates the aberrant growth, body composition, and behavioral phenotypes [135]. Prevention therapies, including ghrelin analogues, have been clinically tested and proven to significantly decrease the appetite in PWS patients [122]. Thus far, no treatment can rescue the full phenotype seen in patients.

As an increasing amount of studies are pointing towards SNORD116 to be the main causative player in PWS, reactivating it would be of interest as a possible therapeutic strategy [136]. Demonstrated by Cruvinal et al. (2014) the histone H3 lysine 9 (H3K9) methyltransferase SETDB1 together with the zinc finger protein ZN274 form a complex that silences the maternal SNORD116 cluster. Upon knockdown of SETDB1 in PWS induced pluripotent stem cells (iPSCs), these cells not only decreased the repressive H3K9me3 mark at the site but also partially restored the maternal 116HG RNA levels in the cells. In addition, knockdown of SETDB1 also disrupted the DNA methylation present on the PWS-IC, shifting it towards a paternal expression pattern [136]. Although a promising approach, the knockdown of SETDB1 lacks specificity as the SNORD116 cluster is not its only histone methylation target [137]. To consider the SETDB1 knockdown/out, it needs to acquire specificity. In another study, Kim et al. (2017) produced a knockout of the ZNF274 gene in neurons derived from PWS iPSCs and rescued SNORD116 expression without it affecting the methylation status in the PWS-IC [138].

Screenings of small molecule compounds possibly able to activate PWS candidate genes have been carried out in mouse fibroblasts derived from the transgenic SNRPN-EGFP mouse. Two inhibitors, UNC0642 and UNC0638, were able to inhibit the histone H3K9 methyltransferase G9a/EHMT2 and activate SNORD116 amongst other genes and subsequently rescued the perinatal lethality seen in this mouse model [139].

This taken together, activation of maternal SNORD116 would pose as a suitable candidate for PWS treatment strategy. To further narrow down the off-target effects, the modified CRISPR-Cas systems employ specificity and are considered to be largely tolerated. As discussed by Wang (2019), the dCas9 tethered with LSD1—a H3K9 demethylase, would specifically reduce the repressive histone marks and allow for SNORD116 reactivation. As an option, manipulation of SETDB1 and ZNF274 could be considered although they could arguably lead to a more global effect [124]. However, in the experiments with ZNF274 and SETDB1 knockdown, the increase of maternally reactivated genes were far from being as expressed as on the paternal allele. The open question is whether a partial restoration is enough to rescue the phenotype. In addition, the experiments were conducted on iPSCs which reflects the conditions during early development, but it does not address the critical windows for restorations later on [136]

### 3.3. Transient Neonatal Diabetes Mellitus Type 1

**Figure 5:**
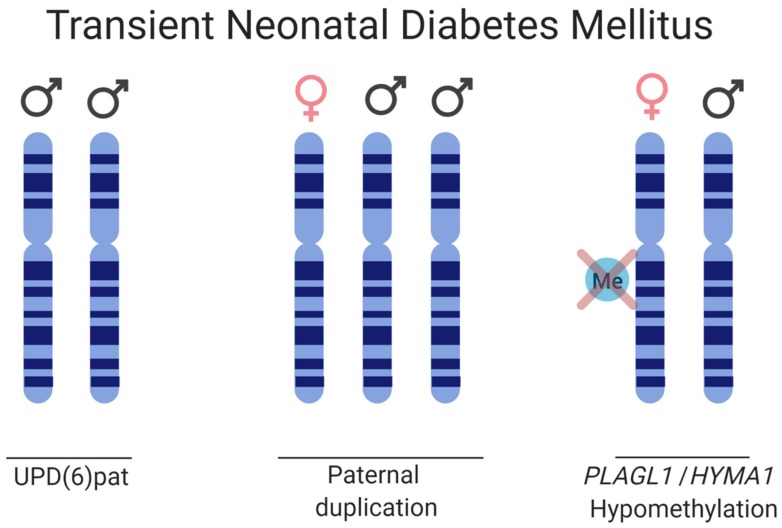
Schematic of the disease causes for TNDM. Three etiologies are depicted, paternal UPD of chromosome 6, paternal duplication of chromosome 6, and hypomethylation on the maternal HYMA1/PLAGL1 promoter, thus allowing for biallelic expression.

Transient neonatal diabetes mellitus type 1 (TNDM) is a rare ID affecting 1:400,000 births. TNDM is characterized by intrauterine growth retardation, failure to thrive in the neonatal stage, dehydration, macroglossia, umbilical hernia, and hyperglycemia requiring exogenous insulin for approximately three months after birth [140]. Endogenous levels of insulin are extremely sparse; however, by 18 months of age, the affected individuals have recovered [141]. Given appropriate treatment and recovery, this rare imprinting disease gives transient phenotypes, but there is a relapse rate of 40% later in life with type 2 diabetes, typically during adolescence. The affected, although diagnosed with type 1 diabetes in infancy, exhibits a type 2 diabetes profile if relapse occurs, as the affected does not have islet cell antibodies characteristic of the immunogenic type I diabetes, nor do they have HLA haplotypes which are diabetes susceptible. A region of 5.4 Mb on 6q24 that is subjected to differential methylation has been linked to TNDM [142]. Genetic and epigenetic etiologies causing TNDM can be grouped as follows: (i) paternal UPD; (ii) interstitial duplications of the paternal locus; and (iii) hypomethylation of the maternal allele allowing for transcription from a normally silenced allele (Figure 5) [143,144]. Analysis of the TNDM region identified two genes which, when overexpressed, cause TNDM, namely; PLAGL1 (pleomorphic adenoma gene-like 1), a zinc finger protein coding gene and HYMAI, an untranslated mRNA [145,146,147]. The maternal imprinting of the genes is modulated by methylation of the promoter/exon 1 of HYMAI, previously shown to be crucial for controlling gene expression in the TNDM mouse model [147].

The PLAGL1 is a zinc finger transcription factor shown to control cell cycle regulation and apoptosis [148]. Furthermore, in mice, the PLAGL1 was shown to positively and negatively regulate beta-cell proliferation and glucose-stimulated insulin release through binding the promoter of the pituitary adenylate cyclase-activating polypeptide type 1 receptor (PACAP) [149]. The role of imprinted transcript HYMA1 is yet to be elucidated [150].

The first-line treatment for TNDM caused by chromosome 6 aberrations is exogenous insulin administered to the patient during the neonatal stage to manage the glucose levels [151]. Upon relapse, however, the consensus on treatment is lacking. In one patient case described by Zhang (2015), a Chinese teenager with a clinical history of maternal hypomethylation at 6q24 associated-TNDM was successfully treated with sulfonylurea, a class of organic compounds used as antidiabetic drugs for type 2 diabetes. The patient reached the glycemic goal of 7–10 mmol/L after sulfonylurea therapy with no subsequent organ damage or apparent side effects [152]. The drug acts by stimulating the release of insulin from the beta cells in the pancreas and can be administered given that the patient has functional receptors for sulfonylurea in the pancreatic beta cells. The rationale behind sulfonylurea treatment is that the patient would have a reduced sensitivity to glucose and its cellular uptake would be aided by sulfonylurea induced insulin secretion [153]. Treatment with insulin, however, is merely a treatment of existing symptoms, not a cure per se and is of little benefit to address other complications connected to the disease. Ideally, a genetic therapy that decreases the PLAGL1 and HYMA1 expression would be early onset treatment. As aforementioned, dCas9/gRNA complexes can be targeted to the promoter sequence and sterically block gene expression, with an efficiency of 60–80% in eukaryotes [69]. For higher silencing efficiencies, the dCas9 and KRAB domains would be employed. As for patients with paternal UPD or interstitial duplications a full silencing would not be beneficial, rather a decrease to mimic the wild-type situation should be considered. For patients harboring hypomethylation on the maternal allele, the dCas9-DNMT3A poses as a convenient candidate for methylating the promoter/exon 1 of HYMA1 and so decreasing the expression to the ordinary levels. The question when and how to therapeutically intervene remains as the TNDM is normally diagnosed after the first symptoms appears. Furthermore, silencing sterically or silencing through the KRAB effector domain is merely transient and more information must be obtained regarding the mode and frequency of administration.

### 3.4. Silver–Russell Syndrome

**Figure 6:**
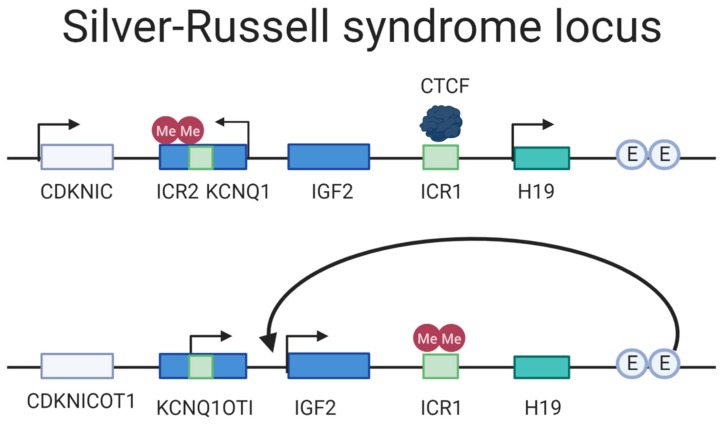
Locus overview of the SRS region. The upper drawing depicts the maternal regulation of the locus. The ICR2 is hypermethylated where the maternally expressed genes KCNQ1 and CDKNIC are expressed. The ICR1 is not methylated, allowing the CTCF motif to bind it and hindering the enhancers (E) to activate IGF2 expression. The lower drawing paternally inherited ICR1 is methylated, thus inhibiting CTCF binding and allowing the enhancers to regulate IGF2 transcription. The ICR2 is not methylated paternally and the non-coding transcript KCNQ1OTI is expressed. Adapted from: Azzi et al. (2009) [158].

Silver–Russell Syndrome (SRS) is a rare ID caused by either (i) maternal UPD of chromosome 7, (ii) maternal UPD of the 11p15 locus, or (iii) paternal hypomethylation of the paternal H19/IGF2 DMR [154]. The disease manifestation varies greatly from very mild to severe phenotypes [155]. The primary locus of interest in SRS is the imprinted region at 11p15, a region with two imprinting domains consisting of the H19/IGF2 IG-DMR (ICR1) and KCNQ1OT1 TSS-DMR (ICR2) regulating the genes H19/IGF2 and the KCNQ1/CDKN1C, respectively [155,156,157]. The H19/IGF2 IG-DMR is paternally methylated, which in turns hinders the CTCF from binding it; this allows the shared enhancers to activate the transcription of IGF2 that is essential for fetal growth (Figure 6) [158]. KCNQ1OT1 TSS-DMR is maternally methylated, and loss of imprinted mutation is the most prevalent cause of Beckwith–Wiedemann syndrome (BWS), a syndrome considered as a distinct but mirrored syndrome of SRS, as they are both caused by aberrations on the 11p15 locus but from different parent-of-origin and have opposite phenotype in disturbed growth [159]. The most frequent cause of SRS is hypomethylation of the H19/IGF2 IG-DMR, displayed by 40% of the affected, in turn leading to a decreased expression of IGF2 and a biallelic expression of H19 [38]. Ten percent has maternal UPD of the chromosome 7 and additional 1–3% have a duplication of the maternally inherited 11p15; for an overview of the locus arrangement see Figure 6. The phenotypes vary drastically in severity, from nearly undetectable symptoms to severe clinical manifestations [160]. The predominant symptoms characterizing SRS is reduced intrauterine and postnatal growth but can also often include macrocephaly and a prominent forehead. In addition, speech delays, organ asymmetry, hypoglycemia, and feeding difficulties are amongst symptoms that are part of the disease picture [161].

Currently, the treatment of SRS aims to manage the symptoms for the patients that vary depending on how the disease manifests itself. Growth hormone therapy, nutritional and caloric supplements and therapy for oral-motor problems may be included as treatments. Moreover, in cases of cleft palate or micrognathia should be addressed by craniofacial surgery [162]. Aforementioned treatments do not provide an entirely satisfactory rescue of all symptoms. An introduction of a mutant ICR in maternal duplication distal chromosome 7 mouse fetuses resulted in activated IGF2 and H19 correction. Furthermore, this reinstatement resulted in significant growth enhancement [163]. Future efforts for correcting the disease could include epi-editors for IGF2 activation, or inhibition through methylation of H19/IGF2 IG-DMR locus to block CTCF from binding and, hence, allow for IGF2 expression.

## 4. Conclusions

The new advancement of CRISPR/Cas9 epigenome editors provides promising tools for editing and possibly treating rare IDs. Dead Cas9 fused or bound to an ‘epi- effector’ domain can modulate gene expression in most conceivable ways able to ameliorate or treat rare IDs caused by aberrant gene expression of imprinted genes. Thus, the epigenome editors based on CRISPR/Cas system represents new ways of activating existing but silenced alleles on the other parent-of-origin chromosome or ways to decrease the expression of genes, which are improperly biallelically expressed. However, the therapeutic potential of the systems reviewed is limited as efficient delivery systems need to be further developed. Additional investigation is also required to identify critical therapeutic window for every individual disease. Albeit many challenges are ahead, the CRISPR systems have beyond doubt opened up novel door for treatment of rare IDs based on epigenome editing, which has not been possible before.

## Figures and Tables

**Table 1:** An overview of the chromosomal regions and frequency of mutations/epimutations for four selected rare ID.

Imprinting Disorder	Chromosome/Gene	Mutation/Epimutation	Frequency
Angelman Syndrome	15q11.2-13q	Maternal deletion	1:12,000/1:20,000
		UPD(15)Pat	
		Methylation defects	
	Ube3a	Point mutations	
Prader-Willi Syndrome	15q11.2-13q	Paternal deletion	1:10,000/1:25,000
		UPD(15)Mat	
		Methylation defects	
Transient Neonatal Diabetes Mellitus	6q24, PLAGL1/HYMA1	UPD(6)Pat	1:400,000
		Paternal duplication	
		Methylation defects	
Silver-Russell Syndrome	7	UPD(7)Mat	1:75,000/1:100,000
	11p15	UPD(11p15)Mat	
		Maternal duplication	
	IGF2/H19	Paternal hypomethylation	
